# Human CD8^+^ T Cells Exhibit a Shared Antigen Threshold for Different Effector Responses

**DOI:** 10.4049/jimmunol.2000525

**Published:** 2020-08-17

**Authors:** Enas Abu-Shah, Nicola Trendel, Philipp Kruger, John Nguyen, Johannes Pettmann, Mikhail Kutuzov, Omer Dushek

**Affiliations:** * Sir William Dunn School of Pathology, University of Oxford, Oxford OX1 3RE, United Kingdom; and; † Kennedy Institute of Rheumatology, University of Oxford, Oxford OX3 7FY, United Kingdom

## Abstract

T cells recognizing cognate pMHC Ags become activated to elicit a myriad of cellular responses, such as target cell killing and the secretion of different cytokines, that collectively contribute to adaptive immunity. These effector responses have been hypothesized to exhibit different Ag dose and affinity thresholds, suggesting that pathogen-specific information may be encoded within the nature of the Ag. In this study, using systematic experiments in a reductionist system, in which primary human CD8^+^ T cell blasts are stimulated by recombinant peptides presented on MHC Ag alone, we show that different inflammatory cytokines have comparable Ag dose thresholds across a 25,000-fold variation in affinity. Although costimulation by CD28, CD2, and CD27 increased cytokine production in this system, the Ag threshold remained comparable across different cytokines. When using primary human memory CD8^+^ T cells responding to autologous APCs, equivalent thresholds were also observed for different cytokines and killing. These findings imply a simple phenotypic model of TCR signaling in which multiple T cell responses share a common rate-limiting threshold and a conceptually simple model of CD8^+^ T cell Ag recognition, in which Ag dose and affinity do not provide any additional response-specific information.

## Key Points

- CD8^+^ T cells produce TNF-α, IL-2, and IFN-γ with similar Ag thresholds.
- Costimulation decreases Ag thresholds similarly for different cytokines.
- A common rate-limiting switch downstream of the TCR can explain these findings.

## Visual Abstract

**Figure:**
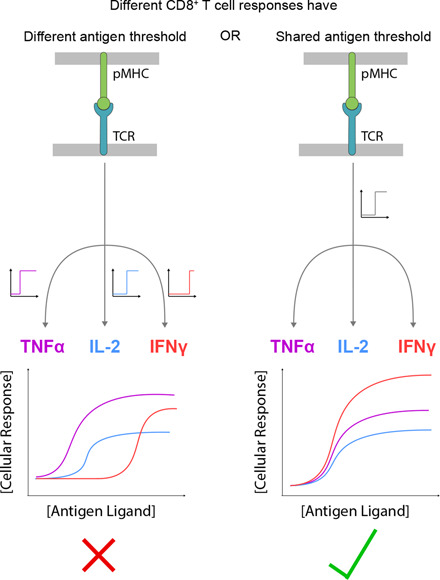

## Introduction

**FIGURE 1.**
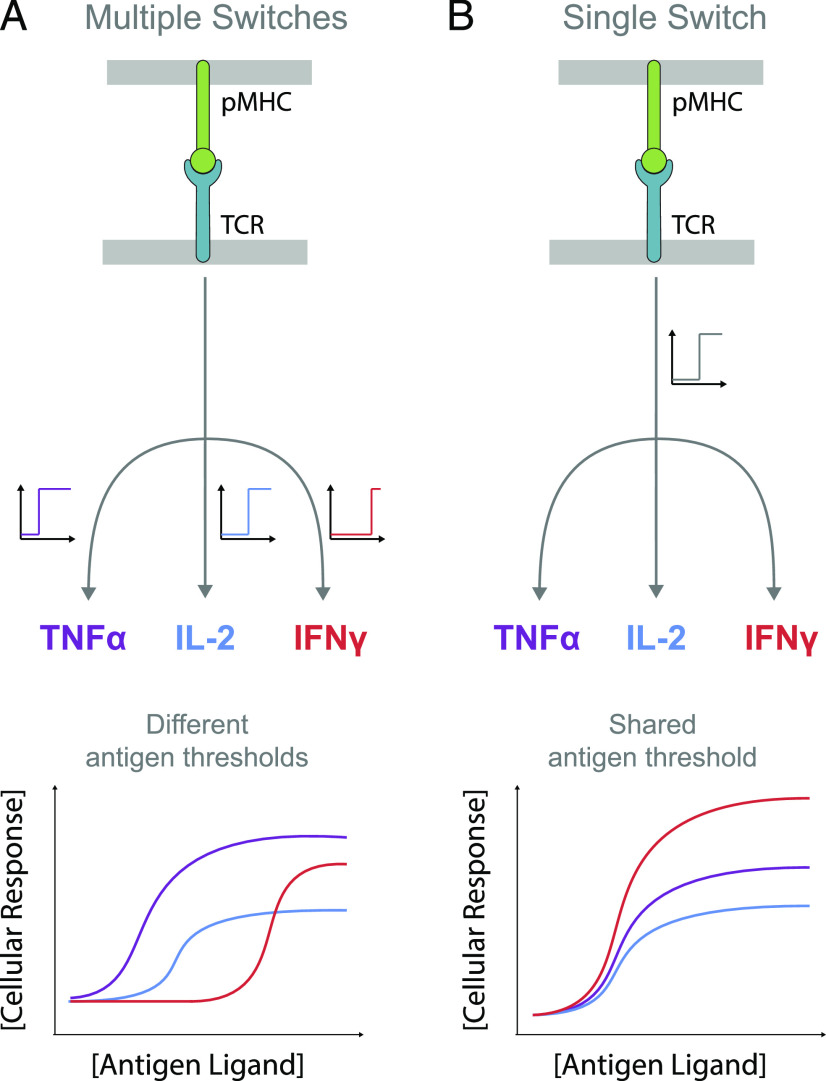
Phenotypic models for pMHC Ag-induced digital TCR signaling leading to multiple T cell effector responses. (**A**) Schematic of TCR signaling showing different rate-limiting digital switches (on/off) controlling different cytokines. The Ag threshold for producing each cytokine can vary if the digital switch has a different threshold with respect to TCR signaling. (**B**) Schematic of TCR signaling showing a common rate-limiting digital switch controlling different cytokines. The Ag threshold is identical because all cytokines are produced when the switch is on. In both models, the amount of cytokine produced can be regulated differently for each cytokine downstream of the switch.

The activation of T cells is critical for immune responses that target infectious agents and tumors. T cells recognize Ags in the form of peptides presented on MHC (pMHC) on professional APC and, in the case of CD8^+^ T cells, also directly on abnormal infected or malignant cells. To eradicate abnormal cells, they initiate a spectrum of effector responses such as direct target cell killing and production of a myriad of cytokines (1). Cytokines are important effectors that mediate cellular communication to promote inflammation (e.g., IFN-γ and TNF-α), proliferation (e.g., IL-2), recruitment of other immune cells (e.g., MIP1β), and interference with viral replication (e.g., IFN-γ), along with many other functions (2–4). Given their critical function in cellular communication, it has been postulated that different cytokines exhibit a different Ag threshold for production (Fig. 1A).

Previous studies presented conditions in which different T cell effector responses appeared to have different pMHC Ag thresholds (5, 6) and are differentially regulated by pMHC affinity and costimulatory molecules (7, 8). Moreover, different T cell clones exhibit a different hierarchical organization of thresholds (9), implying that they have differential wiring of their signaling machinery. Those suggestions have strong implications to the immune response whereby T cells would be able to infer specific information from Ags generated by different pathogens. These studies often relied on peptide-pulsed APCs, which although providing a physiological stimulus, have the caveat that the Ag concentration and stability over time are difficult to control. This may produce apparently different thresholds if the kinetics of each response differ. Moreover, variation in the expression of ligands for cosignaling receptors on T cells can exist over time and differ between experiments. To our knowledge, systematic analyses controlling for these factors have yet to be performed.

T cell responses can exhibit a hierarchy in the Ag threshold concentration and/or affinity if different effector responses exhibit different threshold sensitivities to TCR signaling. Given that TCR signaling is thought to be digital on the single-cell level (10–12) [i.e., individual T cells exhibit an all-or-none signaling response whereby the activation state of downstream signaling is either all active or all inactive (13, 14)], one mechanism to produce different Ag thresholds would be to invoke a different rate-limiting switch for each response that has a different sensitivity to TCR signaling. In contrast, if different responses shared a common rate-limiting switch, then different responses would share a comparable Ag threshold. In the latter model, Ag affinity would control the threshold Ag concentration comparably for all responses. In both models, the production of cytokine can be regulated downstream of the switch so that the temporal kinetics and magnitude of the response (e.g., the amount of cytokine) can be different for different cytokines.

In this study, we systematically stimulate primary human CD8^+^ T cell blasts in a reductionist system that allows for the precise control of pMHC Ag dose and affinity. We find that although Ag affinity controls the Ag dose threshold for inducing cytokine production, the threshold is comparable for different cytokines across a wide range of affinities. By incorporating ligands to CD28, CD2, and CD27, we show that although they increase cytokine production, they do so similarly for different cytokines so that the threshold remains comparable. Finally, we reproduce these findings in a recently described experimental system (15) that allows for the study of quiescent primary memory CD8^+^ T cells responding to autologous monocyte-derived dendritic cells. The work suggests a conceptually simpler phenotypic model for TCR signaling with implications for the role of Ag dose and affinity in mediating T cell responses.

## Materials and Methods

### Protein production

pMHCs were refolded in vitro from the extracellular residues 1–287 of the HLA-A*02:01 α-chain, β2-microglobulin, and NY-ESO-1_157_*_−_*_165_ peptide variants as described previously (16). CHO cell lines permanently expressing the extracellular part of human CD86 (aa 6–247) or human CD58 (aa 29–213) with a His tag for purification and a BirA biotinylation site were kindly provided by Simon Davis (University of Oxford, Oxford, U.K.). Cells were cultured in glutamine synthetase selection medium and passaged every 3–4 d. After four to five passages from thawing a new vial, cells from two confluent T175 flasks were transferred into a cell factory and incubated for 5–7 d, after which the medium was replaced. The supernatant was harvested after another 3 wk, sterile filtered, and dialyzed overnight. The His-tagged protein (CD86 or CD58) was purified on a Nickel-NTA Agarose column.

CD70 (CD27 ligand) expression constructs were a kind gift from Harald Wajant (Würzburg, Germany) and contained a Flag tag for the purification and a tenascin-C trimerization domain. We added a BirA biotinylation site. The protein was produced by transient transfection of HEK 293T cells with X-tremeGENE HP Transfection Reagent (Roche), according to the manufacturer’s instructions, and purified following a published protocol (17), with the exception of the elution step in which we used acid elution with 0.1 M glycine–HCl at pH 3.5.

The pMHC or costimulatory ligand was then biotinylated in vitro by BirA enzyme, according to the manufacturer’s instructions (Avidity Biosciences), purified using size-exclusion chromatography with HBS-EP (0.01 M HEPES [pH 7.4], 0.15 M NaCl, 3 mM EDTA, and 0.005% v/v Tween 20) as flow buffer, and stored in aliquots at −80°C.

### Production of lentivirus for transduction

HEK 293T cells were seeded into six-well plates before transfection to achieve 50–80% confluency on the day of transfection. Cells were cotransfected with the respective third-generation lentiviral transfer vectors and packaging plasmids using Roche X-tremeGENE 9 (0.8 μg lentiviral expression plasmid, 0.95 μg pRSV-rev, 0.37 μg pVSV-G, 0.95 μg pGAG). The supernatant was harvested and filtered through a 0.45-μm cellulose acetate filter 24–36 h later. The 1G4 TCR used for this project was initially isolated from a melanoma patient (18). The affinity maturation to the c58c61 variant used in this study (referred to as 1G4^Hi^) was carried out by Adaptimmune (19). The TCR in this study has been used in a standard third-generation lentiviral vector with the EF1α promoter.

### T cell isolation and culture

Up to 50 ml peripheral blood was collected by a trained phlebotomist from healthy volunteer donors after informed consent had been taken. This project has been approved by the Medical Sciences Interdivisional Research Ethics Committee of the University of Oxford (R51997/RE001), and all samples were anonymized in compliance with the Data Protection Act. Alternatively, leukocyte cones were purchased from National Health Services Blood and Transplant service. Only HLA-A2^−^ peripheral blood or leukocyte cones were used because of the cross-reactivity of the high-affinity receptors used in this project, which leads to fratricide of HLA-A2^+^ T cells (20). CD8^+^ T cells were isolated directly from blood using the CD8^+^ T Cell Enrichment Cocktail (STEMCELL Technologies) and density gradient centrifugation according to the kit’s instructions. The isolated CD8^+^ T cells were washed and resuspended at a concentration of 1 × 10^6^ cells per milliliter in completely reconstituted RPMI 1640 supplemented with 50 U/ml IL-2 and 1 × 10^6^ CD3/CD28-coated Human T-Activator Dynabeads (Life Technologies) per milliliter. The next day, 1 × 10^6^ T cells were transduced with the 2.5 ml virus-containing supernatant from one well of HEK cells supplemented with 50 U/ml of IL-2. The medium was replaced with fresh medium containing 50 U/ml IL-2 every 2–3 d. CD3/CD28-coated beads were removed on day 5 after lentiviral transduction, and the cells were used for experiments on days 10–14. TCR expression was assessed by staining with NY-ESO 9V PE–conjugated tetramer (in-house produced using refolded HLA*A0201 with NY-ESO 9V and streptavidin–PE [Bio-Rad AbD Serotec or BioLegend]) using flow cytometry.

### T cell stimulation

T cells were stimulated with titrations of plate-immobilized pMHC ligands with or without coimmobilized ligands for accessory receptors. Ligands were diluted to the working concentrations in sterile PBS. Fifty microliters serially 2-fold diluted pMHC were added to each well of high-binding capacity of streptavidin-coated 96-well plates (15500; Thermo Fisher Scientific). After a minimum 45-min incubation at room temperature, the plates were washed once with sterile PBS. Where accessory receptor ligands were used, those were similarly diluted and added to the plate for a second incubation of 45–90 min. After washing the stimulation plate with PBS, 7.5 × 10^4^ T cells were added in 200 μl complete RPMI without IL-2 to each stimulation condition. The plates were spun at 50–200 × *g* for 2 min to settle down the cells and then incubated at 37°C with 5% CO_2_ for 8 h.

### T cells–Ag-presenting cells stimulation assay

The assay was performed as previously described (15). Briefly, memory CD8^+^ T cells were isolated from anonymized leukapheresis products obtained from the National Health Services at Oxford University Hospitals by (REC 11/H0711/7) using Memory CD8^+^ Isolation Kit (STEMCELL Technologies). T cells were harvested and washed three times with Opti-MEM (Life Technologies). The cells were resuspended at 25 × 10^6^/ml, and 2.5–5 × 10^6^ cells were mixed with the desired mRNA products and aliquoted into 100–200 μl per electroporation cuvette (Cuvette Plus, 2 mm gap; BTX Technologies). For each 10^6^ CD8^+^ T cells, 5 μg of each TCRα, TCRβ, and CD3ζ RNA was used. Cells were electroporated at 300 V for 2 ms in an ECM 830 Square Wave Electroporation System (BTX Technologies). Cells were used after 24 h. Monocytes were enriched from the same leukapheresis products using RosetteSep kits (STEMCELL Technologies) and were then cultured at 1–2 × 10^6^/ml in 12-well plates with differentiation media containing 50 ng/ml IL-4 (PeproTech) and 100 ng/ml granulocyte-monocyte CSF (ImmunoTools) for 24 h. For maturation, the following cytokines were added for an additional 24 h: 1 μM PG E2 (PGE2; Sigma), 10 ng/ml IL-1β (Bio-Techne), 50 ng/ml TNF-α (PeproTech), and 20 ng/ml IFN-γ (Bio-Techne). Monocyte-derived dendritic cells were loaded with peptide for 90 min at 37°C. T cells and dendritic cells were mixed at 1:1 ratio, 50,000 cells each, and incubated for 6 or 24 h before supernatant was collected and analyzed.

### ELISA

Supernatants from stimulation experiments were used fresh. Cytokine concentrations were measured by ELISAs according to the manufacturer’s instructions in Nunc MaxiSorp flat-bottom plates (Invitrogen) using Uncoated ELISA Kits (Invitrogen) for TNF-α, IFN-γ, and IL-2. Lactate dehydrogenase (LDH) Cytotoxicity Detection Kit (Takara Bio) was used as per manufacturer instructions to detect cell killing.

### Data analysis

A smoothing function with 10,000 data points was fitted to the experimental data to empirically extract the maximum amount of cytokine produced across different pMHC concentrations [maximum efficacy (E_max_)] and the pMHC concentration producing an EC_50_ for each dose-response curve. The difference of two sigmoidal dose-response curves was used as a smoothing function to capture the bell-shaped dose-response curve frequently observed (16), and it was fitted to the experimental data using the *lsqcurvefit* function in MATLAB (The MathWorks).

**FIGURE 2.**
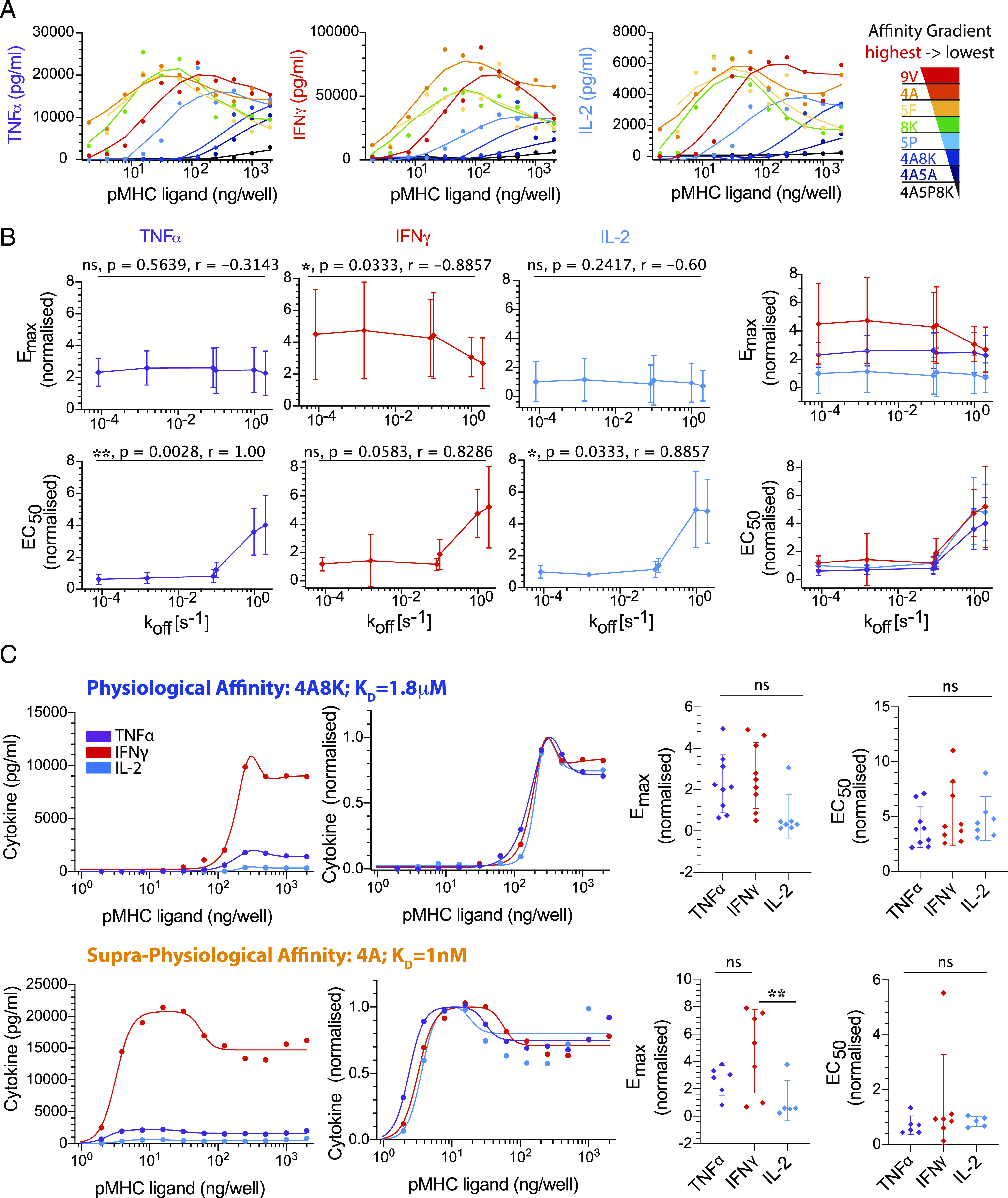
Different cytokines exhibit a comparable Ag dose threshold over a large variation in Ag affinity. (**A**) Representative data showing supernatant TNF-α, IFN-γ, and IL-2 over pMHC dose for different pMHC affinities (colors). (**B**) Fitted E_max_ (top row) and EC_50_ (bottom row) for each cytokine individually (left three columns) or overlaid (right column) over the TCR/pMHC off-rate (*k*_off_) measured at 37°C (Supplemental Fig. 1A). Reliable estimates were not possible for the two lowest affinity pMHCs (4A5A and 4A5P8K), and they were omitted from the quantitative analysis. (**C**) Representative overlay of TNF-α, IFN-γ, and IL-2 directly (first column) or normalized by their respective E_max_ value (second column) for a physiological (top row) and supraphysiological (bottom row) affinity pMHC. Dot plots for E_max_ (third column) and EC_50_ (fourth column) show the data across nine independent biological repeats with different donors. This analysis highlights that within the resolution of our 2-fold dilutions in pMHC dose, no significant difference is observed between the EC50 threshold for different cytokines. ANOVA corrected for multiple comparisons by Tukey test (**p = 0.002). The normalized dose-response curves for all nine independent repeats is displayed in Supplemental Fig. 2. Error bars are SD of mean. Solid lines in representative datasets are the fits used to extract Emax and EC50. Normalization and data fitting is described in *Materials and Methods*.

**FIGURE 3.**
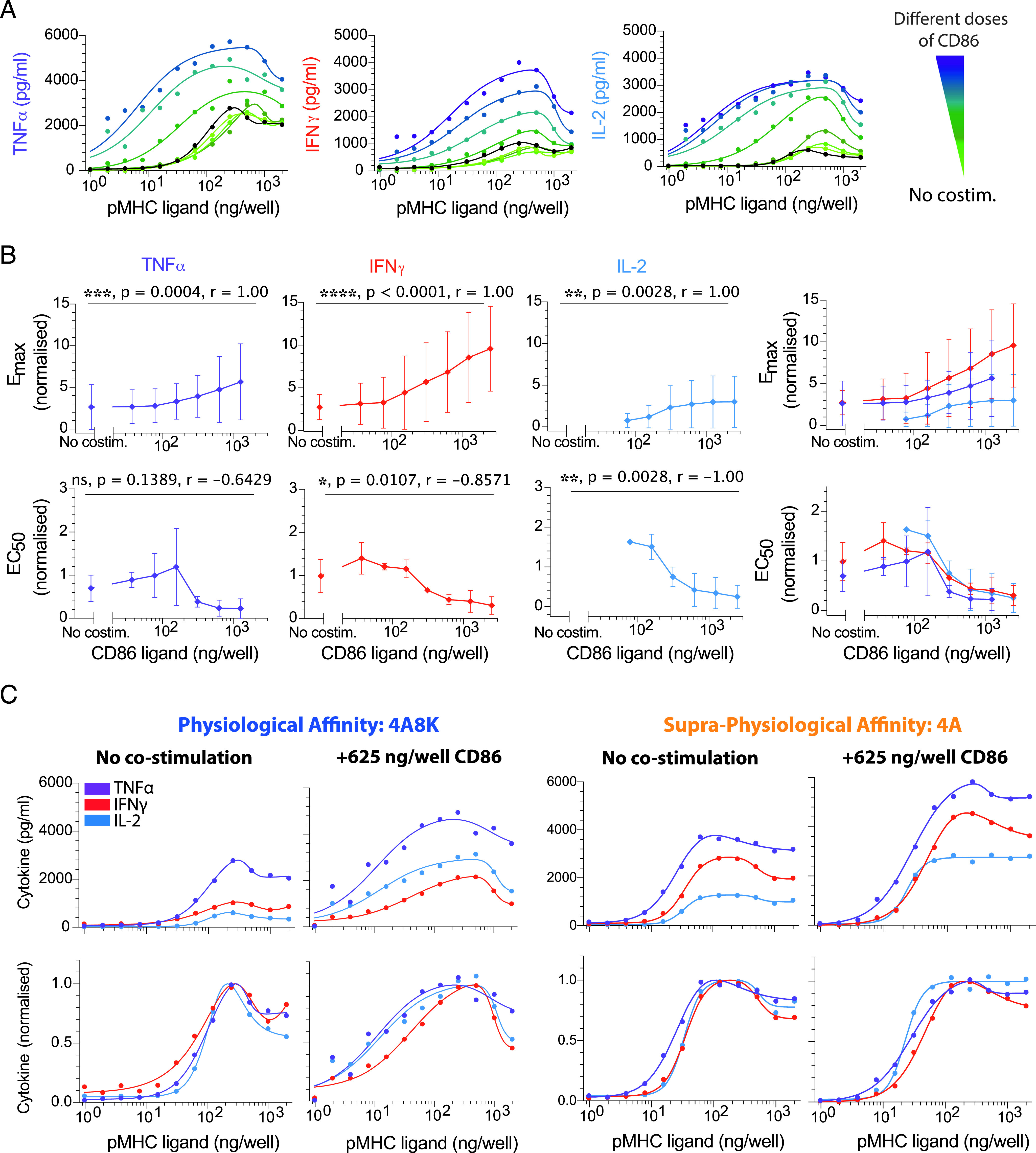
CD28 costimulation decreases the Ag threshold for cytokine production comparably for different cytokines. (**A**) Representative data showing secretion of TNF-α, IFN-γ, and IL-2 over the pMHC dose (physiological affinity, 4A8K) when T cells were costimulated with different doses of CD86 (colors). Black solid line is without costimulation. (**B**) Normalized E_max_ (top row) and EC_50_ (bottom row) for each cytokine over the CD86 dose confirms that costimulation can control both efficacy and potency, respectively. Overlay of E_max_ and EC_50_ for all cytokines (rightmost panels). (**C**) Representative overlay of TNF-α, IFN-γ, and IL-2 directly (top row) or normalized (bottom row) for the indicated pMHC and costimulation condition. The Ag dose threshold for different cytokines is comparable irrespective of CD86 dose. For statistical comparison, see Supplemental Fig. 3. Error bars are SD of mean for three independent donors. Normalization of experimental data are described in *Materials and Methods*. Solid lines in representative datasets are the fits used to extract E_max_ and EC_50_.

**FIGURE 4.**
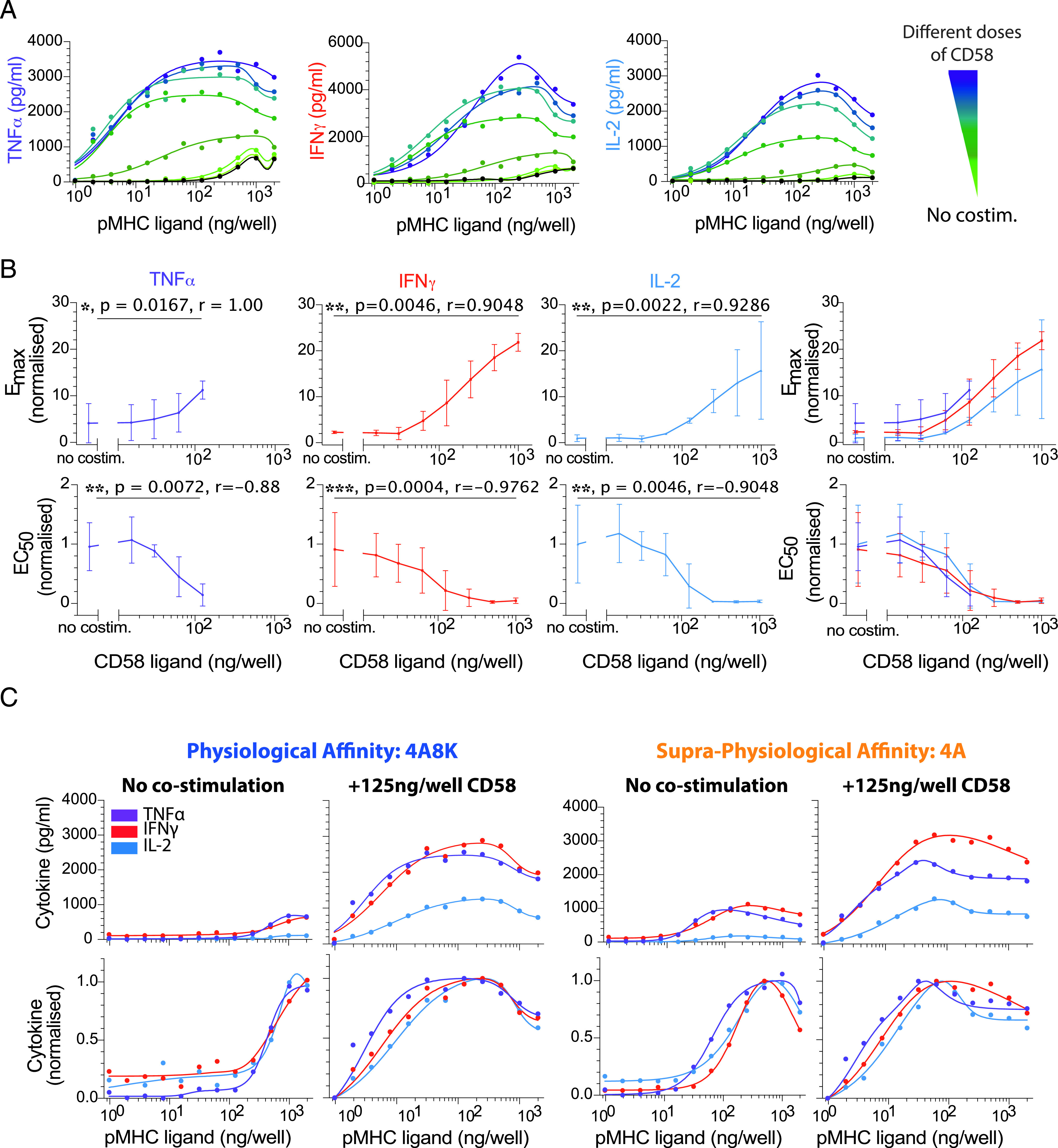
CD2 costimulation decreases the Ag threshold for cytokine production comparably for different cytokines. (**A**) Representative data showing secretion of TNF-α, IFN-γ, and IL-2 over the pMHC dose (physiological affinity, 4A8K) when T cells were costimulated with different doses of CD58 (colors). Black solid line is without costimulation. (**B**) Normalized E_max_ (top row) and EC_50_ (bottom row) for each cytokine over the CD58 dose confirms that costimulation can control both efficacy and potency, respectively. Overlay of E_max_ and EC_50_ for all cytokines (rightmost panels). (**C**) Representative overlay of TNF-α, IFN-γ, and IL-2 directly (top row) or normalized (bottom row) for the indicated pMHC and costimulation condition. The Ag dose threshold for different cytokines is comparable irrespective of CD58 dose. For statistical comparison, see Supplemental Fig. 4. Error bars are SD of mean for three independent donors. Normalization of experimental data are described in *Materials and Methods*. Solid lines in representative datasets are the fits used to extract E_max_ and EC_50_.

**FIGURE 5.**
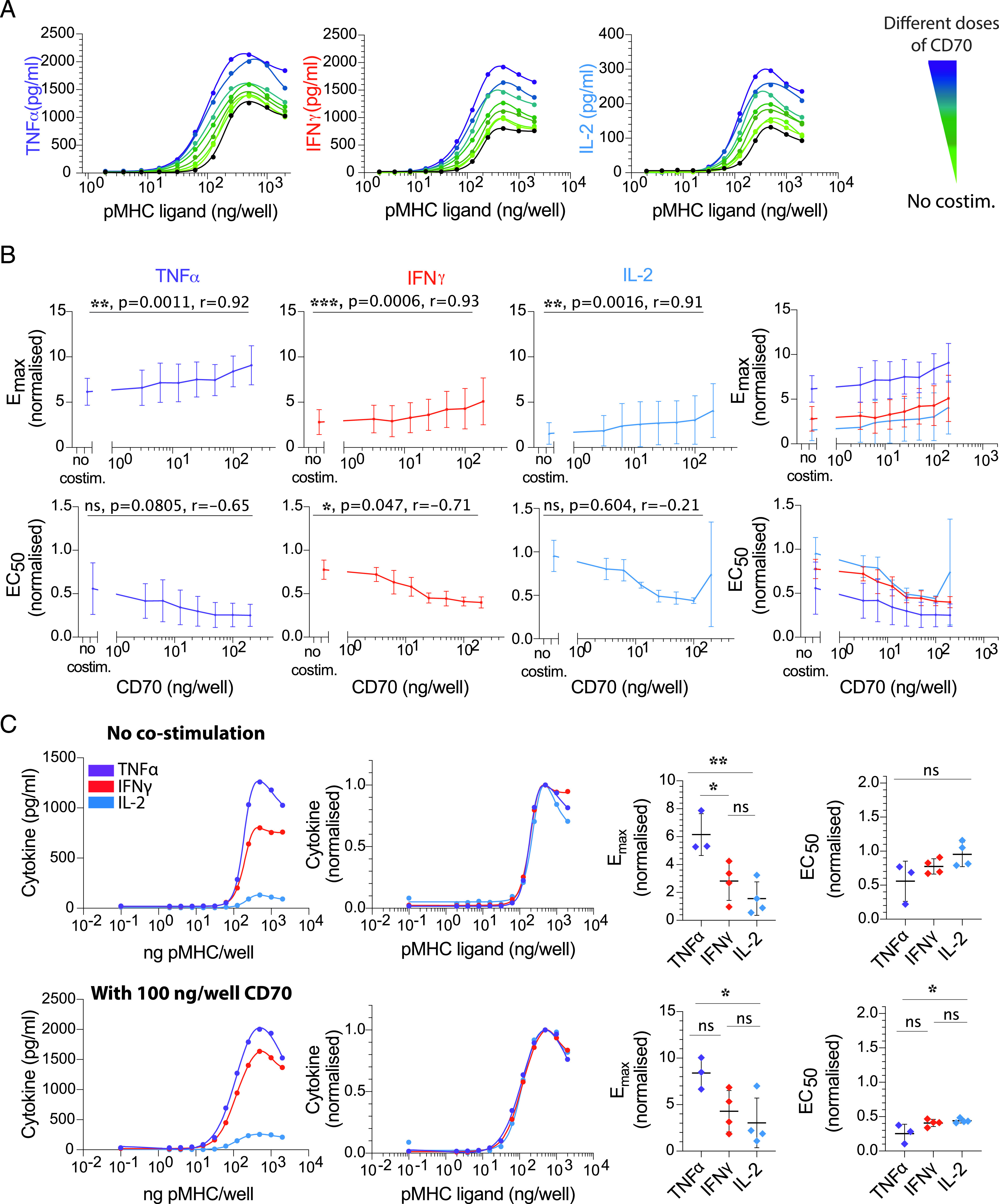
CD27 costimulation decreases the Ag threshold for cytokine production comparably for different cytokines. (**A**) Representative data showing secretion of TNF-α, IFN-γ, and IL-2 over the pMHC dose (physiological affinity, 4A8K) when T cells were costimulated with different doses of trimeric CD70 (colors). Black solid line is without costimulation. (**B**) Normalized E_max_ (top row) and EC_50_ (bottom row) for each cytokine over the CD70 dose confirms that costimulation can control both efficacy and potency, respectively, albeit to a lower extent compared with CD28 and CD2. Overlay of E_max_ and EC_50_ for all cytokines (rightmost panels). (**C**) Representative overlay of TNF-α, IFN-γ and IL-2 directly (first column) or normalized (second column) without costimulation (top row) or with a single dose of CD70 costimulation (bottom row) for 4A8K pMHC. The Ag dose threshold for different cytokines is comparable under both conditions. This conclusion is reflected in the dot plots of E_max_ and EC_50_ showing no significant or up to 2-fold differences between the cytokines in the absence (top row) or presence (bottom row) of CD70. Error bars are SD of mean for three independent donors. Normalization of experimental data are described in *Materials and Methods*. Solid lines in representative datasets are the fits used to extract E_max_ and EC_50_. ANOVA corrected for multiple comparisons by Tukey test (*p = 0.03, **p = 0.0052).

**FIGURE 6.**
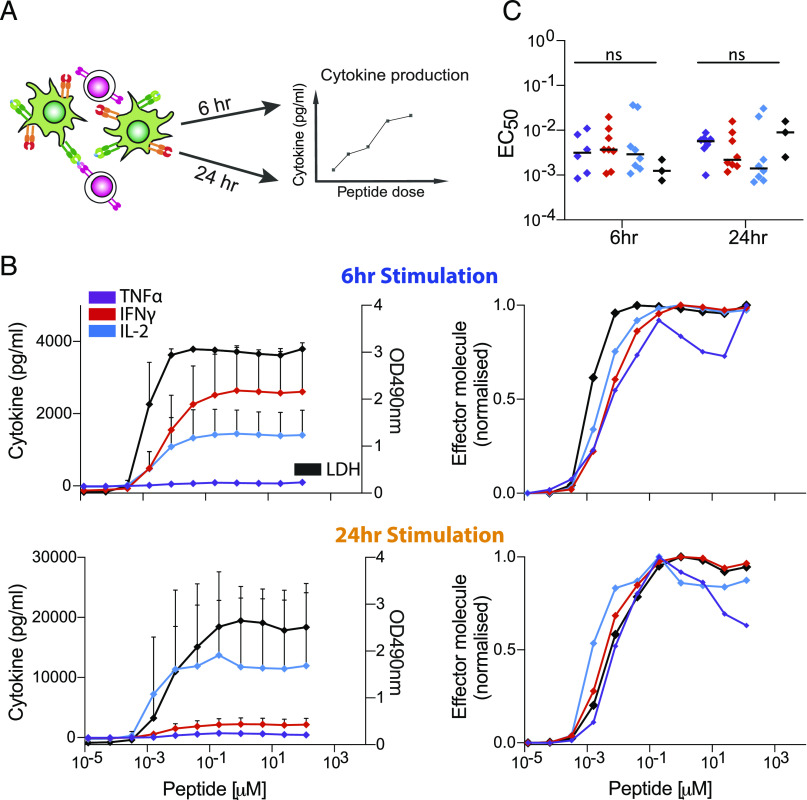
Memory CD8^+^ T cells produce different cytokines and induce killing at a comparable Ag threshold. (**A**) Schematic of experimental assay showing memory CD8^+^ T cells electroporated with the wild-type 1G4 TCR (magenta) recognizing 9V peptides on MHC (orange) loaded onto monocyte-derived dendritic cells (green) for 6 or 24 h before cytokine or LDH levels are measured in the supernatant. The 1G4 TCR affinity for the 9V is *K*_d_ = 7 μM (21). (**B**) Average supernatant TNF-α, IFN-γ, and IL-2 (left *y*-axis) or LDH (right *y*-axis) as a function of peptide concentration following T cell activation by peptide-loaded mature monocyte-derived dendritic cells for 6 h (top) or 24 h (bottom). Error bars are SD of mean from at least six (cytokines) or three (LDH) independent donors. (**C**) The EC_50_ for each effector molecule from multiple donors are plotted at 6 and 24 h (dots are individual donors, and horizontal bar is median), showing no significant difference between the different cytokines (ANOVA corrected for multiple tests using Tukey test).

Experiments with human donors are often highly variable, and we found large quantitative differences in the range of cytokine production between experimental repeats. Before averaging across different donors in Figs. 2B, 3B, 4B and 5B, the E_max_ and EC_50_ were thus, for each donor, normalized to the mean E_max_ and mean EC_50_, respectively, for all the cytokines across all pMHC affinities (Fig. 2, Supplemental Fig. 1) or all doses of the costimulatory ligand (Figs. 3–5, Supplemental Figs. 2–4). The normalized E_max_ and EC_50_ were then averaged across all human donors, and they are presented in Figs. 1B, 2B, 3B, 4B, 5B and 6B as a fold change relative to the respective metric for IL-2 in response to the highest-affinity pMHC in Fig. 2 or the experimental control with pMHC alone in Figs. 3–5 and Supplemental Figs. 1–4 to allow for a more intuitive comparison of cytokines.

Similarly, before averaging across different donors for dot plots in each figure, the E_max_ and EC_50_ were, for each donor, normalized to the mean E_max_ and mean EC_50_, respectively, for any given pMHC affinity (Fig. 2) or dose of the costimulatory ligand (Figs. 3–5, Supplemental Figs. 3, 4). The normalized E_max_ and EC_50_ were then averaged across all human donors, and they are again presented in Supplemental Figs. 1–4 as a fold change relative to the respective metric for IL-2 to more directly identify potential differences in E_max_ and EC_50_ between cytokines at each pMHC affinity and concentration of costimulatory ligand.

### Statistical analysis

A nonparametric Spearman correlation test was used to identify whether pMHC affinity (Fig. 2) or the concentration of costimulatory ligands (Figs. 3–5, Supplemental Figs. 3, 4) correlated with the E_max_ and EC_50_ of each cytokine response (Figs. 1B, 2B, 3B, 4B, 5B, 6B). Ordinary repeated-measures two-way ANOVA corrected for multiple comparisons by Tukey test was performed on experimental data to determine whether differences in E_max_ and EC_50_ at each pMHC affinity (Fig. 2) or concentration of costimulatory ligand (Figs. 3–5, Supplemental Figs. 2–4) were statistically significant between cytokines. GraphPad Prism was used for all statistical analyses.

## Results

### Different cytokines exhibit comparable Ag dose thresholds over a wide range of affinity

To investigate the Ag threshold required to elicit different effector cytokines and to differentiate between proposed models (Fig. 1), we first used a reductionist system to exclude any contribution from extrinsic factors such as pMHC stability and variation in costimulation ligands on APCs. In this system, primary human CD8^+^ T cell blasts that have been transduced to express the affinity enhanced 1G4 TCR (c58c61) (18, 19) were stimulated by plate-immobilized recombinant pMHC (16, 21, 22, and N.C. Trendel, P. Kruger, J. Nguyen, S. Gaglione, and O. Dushek, manuscript posted on bioRxiv). The use of the c58c61 TCR allowed us to explore cytokine thresholds when T cells are stimulated by a panel of eight pMHCs that span *>*10 million–fold variation in affinity from supraphysiological therapeutic affinities (picomolars) to physiological affinities (micromolars) (Supplemental Fig. 1A, 1B) (16). After 8 h of interacting with plates coated with different concentrations of the different affinity Ags, the production of TNF-α, IFN-γ, and IL-2 was quantified in the supernatants (Fig. 2A).

To compare the Ag threshold of each cytokine, we first determined the E_max_ (the maximum response across all Ag doses) and the Ag potency (E_50_, the threshold concentration of Ag producing 50% of E_max_) by directly fitting the dose-response curves (see *Materials and Methods*). We then plotted representative experiments on the same graph either directly or normalized by the E_max_ of each cytokine to clearly identify whether any differences in Ag threshold were present (Fig. 2C, Supplemental Fig. 1C). This analysis shows that although a different amount of each cytokine is detected, the Ag threshold for inducing these cytokines is comparable and within the resolution of our 2-fold dilutions. This conclusion is reflected in the EC_50_ values for the nine independent biological repeats, showing that no significant difference can be detected (Fig. 2C, Supplemental Fig. 1C, right two panels).

Although we focused on TNF-α, IFN-γ, and IL-2, we found that the same threshold applied for MIP-1β (Supplemental Fig. 2). Therefore, different cytokines exhibit a comparable Ag dose threshold across a wide variation in Ag affinity (*∼*25,000-fold, 9V to 4A8K) when T cell activation is mediated exclusively by pMHC through the TCR.

### Costimulation increases cytokine production but maintains a comparable Ag dose threshold for different cytokines

Given that we observed a comparable Ag dose threshold for different cytokines when stimulating T cells with pMHC Ag alone, we hypothesized that differences between cytokines may emerge when providing T cell costimulation. This hypothesis is motivated by the fact that in previous reports showing differential cytokine thresholds, Ag was expressed on the surface of APC in the context of ligands to various costimulatory receptors on T cells. To directly test this hypothesis, we used our reductionist experimental system to copresent pMHC with a titration of three prominent CD8^+^ T cell costimulatory ligands: CD86 (ligating CD28; Fig. 3), CD58 (ligating CD2; Fig. 4), or CD70 (ligating CD27; Fig. 5).

The ligand CD86 binds the costimulatory receptor CD28 (23). As expected, a titration of CD86 increased the amount of cytokine detected in the supernatant and reduced the Ag dose threshold required to detect cytokine (Fig. 3A, 3B). However, when we directly compared thresholds for individual cytokines, we observed that all responded in a quantitatively similar manner to individual CD86 concentrations across two different pMHCs that have a large variation in affinity (Fig. 3C, Supplemental Fig. 3).

The ligand CD58 binds the costimulatory receptor CD2, which is thought to enhance adhesion (24–26) and intracellular signaling (P. Demetriou, E. Abu-Shah, S. McCuaig, V. Mayya, S. Valvo, K. Korobchevskaya, M. Friedrich, E. Mann, L.Y.W. Lee, T. Starkey, M.A. Kutuzov, J. Afrose, A. Siokis, M. Meyer-Hermann, D. Depoil, and M.L. Dustin, manuscript posted on bioRxiv; Ref. 27).We observed a large ∼50-fold increase in potency (decrease in EC_50_) and a *>*10-fold increase in efficacy (Fig. 4A, 4B). However, as with CD28 costimulation, the Ag dose threshold appeared comparable for different cytokines under all conditions (Fig. 4C). We did observe a moderate difference for the supraphysiological pMHC affinity with a low concentration of CD58, whereby the EC_50_ value of TNFa was 2-fold lower compared with IFN-γ and IL-2 (Supplemental Fig. 4). However, given that this was only apparent under some conditions and similar in magnitude to the resolution of our assay (2-fold pMHC dilutions), it is unclear whether it is biologically relevant (see also *Discussion*).

The ligand CD70 forms trimers that induce trimerization of the costimulatory receptor CD27, which is a member of the TNF family of costimulatory molecules (28). We observed that a titration of recombinant and trimeric CD70 exhibited increased cytokine production (Fig. 5A) with a modest impact on both the efficacy and potency compared with CD2 and CD28 ligation (Fig. 5B). However, as with CD28 and CD2 costimulation, the Ag dose threshold for different cytokines appeared equivalent under all conditions (Fig. 5C).

Taken together, these data suggest that T cell costimulation by three prominent receptors that span diverse families can control cytokine production efficacy and potency but are unable to control Ag potency differently for each cytokine we investigated.

### Memory CD8^+^ T cells exhibit comparable cytokine thresholds in response to autologous APC

Given that we observed comparable cytokine thresholds using T cell blasts in a reductionist system and that limited data are available for human T cells, it was important to determine if these conclusions hold in a more physiological system. To this end, we directly isolated memory CD8^+^ T cells from healthy donors and used RNA electroporation to express the wild-type 1G4 TCR, and in parallel, we generated autologous monocyte-derived dendritic cells that we loaded with a titration of the 9V peptide, which binds with a *K*_d_ of 7.2 μM (21)] before mixing them with T cells for either 6 or 24 h (Fig. 6A). This experimental system has recently been described in detail (15). In contrast to the reductionist system in which ligands are immobile, this system allowed for both Ag and costimulation ligand mobility in the fluid plasma membrane of the APC. We focused on memory CD8^+^ T cells because naive T cells do not produce cytokines on our experimental timescales (29). Consistent with our previous results, we observed a comparable Ag threshold for all cytokines (Fig. 6B, 6C). We were also able to assess the ability of memory cells to kill target cells by measuring the release of LDH into the supernatant, finding an Ag threshold that was comparable to the cytokine threshold.

## Discussion

Using systematic experiments in a reductionist plate-based system, with precise control of pMHC Ag dose/affinity and costimulation through CD28, CD2, and CD27, we found no evidence for different Ag thresholds for different cytokines produced by CD8^+^ T cell blasts. We observed similar results when using memory CD8^+^ T cells stimulated by monocyte-derived APC expressing a combination of cosignaling receptor ligands.

Costimulation by CD2, CD27, and CD28 increased T cell cytokine production in our plate-based reductionist system but were quantitatively distinct (Figs. 3, 4, 5). In all cases, costimulation increased the absolute amount of cytokine produced (increased in E_max_) and increased Ag potency (decreased in EC_50_). However, the fold increase in E_max_ and the fold decrease in EC_50_ were largest for CD2 and not for the more canonical costimulation receptor CD28, whereas CD27 exhibited only modest fold changes. Although CD2 was initially reported to have only a subtle role in T cell activation in mice (30, 31), it is increasingly clear that it is important for human T cell activation (P. Demetriou, E. Abu-Shah, S. McCuaig, V. Mayya, S. Valvo, K. Korobchevskaya, M. Friedrich, E. Mann, L.Y.W. Lee, T. Starkey, M.A. Kutuzov, J. Afrose, A. Siokis, M. Meyer-Hermann, D. Depoil, M.L. Dustin, manuscript posted on bioRxiv and 32, 33) and may be particularly important for CD8^+^ T cells that do not express CD28 (34). Although costimulation in this reductionist system clearly controlled the Ag threshold for T cell cytokine production, it appeared to do so similarly for different cytokines. Therefore, we found no evidence that a different Ag threshold elicited different effector cytokines in CD8^+^ T cells.

The discrepancy between our results and previous work may be a result of differences in experimental assays and time points. We have measured population-level supernatant cytokine levels, whereas previous work relied on intracellular cytokine staining (6, 8, 9), which provides single-cell information but by blocking secretion may affect different cytokines differently. This difference is apparent in a study that directly compared the two methods showing a different threshold for different cytokines when using intracellular staining but not in the supernatant [see Fig. 2 in (20)]. A technically more demanding assay based on single-cell cytokine secretion has shown that a single pMHC can induce both TNF-α and IL-2, implying that their Ag threshold is comparable (35). In addition, it is now clear that different cytokines exhibit different production kinetics (36, 37), and production depends on continuous TCR/pMHC engagement (38). Therefore, the pMHC degradation rate may introduce apparent differences in thresholds with cytokines having faster production kinetics appearing to have a lower threshold. To control for this, we have used a highly stable variant of the NY-ESO-1 peptide Ag (9C to 9V) and used recombinant pMHC for constant presentation (N.C. Trendel, P. Kruger, J. Nguyen, S. Gaglione, and O. Dushek, manuscript posted on bioRxiv).

We highlight that although the Ag threshold for producing different cytokines may be comparable, or indeed identical, there are multiple mechanisms that enable differential regulation. For example, it is clear that there are differences in bulk cytokine production kinetics (36, 37), which may be a result of different mRNA expression and stability (39–41) and sequential production programs (36). Although differences in individual cytokine levels are clearly observed in individual experiments (see representative curves in Figs. 2C, 3C, 4C, and 5C), variability across human donors has meant that these differences were not always statistically significant (see E_max_ values in dot plots). Therefore, although the decision to produce cytokine is tightly coupled to a common Ag threshold, the kinetics of production and the absolute amount can be regulated differently.

Our findings support a molecular signaling model whereby a digital signaling switch is rate-limiting for all cytokines with differences in the absolute amount arising as a result of different production kinetics downstream (Fig. 1B). A digital switch has been reported in the TCR signaling pathway (10–12), and a large number of T cell responses, including cytokine production, have been shown to be digital (14, 35, 42). Therefore, the observation that the induction of different cytokines have a comparable Ag threshold implies that they have comparable TCR signaling thresholds, and it is likely that they share a common rate-limiting switch. As discussed above, different production kinetics can arise from a variety of mechanisms downstream of the rate-limiting switch (e.g., different mRNA stability). If this switch is proximal to the TCR, it would imply that other effector responses share the same Ag threshold as cytokines. In experiments with APCs, we observed a similar Ag threshold between cytokines and a proxy for killing (Fig. 6). This is consistent with the observation that both killing and cytokine production can be observed in response to <5 pMHC per APC (35, 43, 44).

The systematic analysis of multiple T cell responses we have performed suggests that Ag recognition switches “on” CD8^+^ T cell effector functions, implying a common Ag dose threshold, and this common threshold depends on Ag affinity. This model is conceptually appealing because the Ag dose and affinity are chance factors that do not necessarily encode any pathogen-specific information that may favor one effector response over another. In this model, pathogen-specific information may be encoded by TCR-extrinsic factors, such as ligands to other cosignaling receptors (45). This is consistent with recent in vitro (46, 47) and in vivo (48) data showing that different Ag doses and affinities produce CD8^+^ T cells with similar response potentials. This conclusion may differ for CD4^+^ T cell differentiation, in which Ag dose can selectively induce regulatory T cells, Th1, and Th2 phenotypes (49–51). In summary, we propose that T cell effector responses are maintained by a common critical Ag-dependent threshold that can be subsequently regulated by temporal integration and extrinsic cosignaling receptors that can be response specific.

## Supplementary Material

Data Supplement
