# Supplementary material for: Human CD8+ T Cells Exhibit a Shared Antigen Threshold for Different Effector Responses
Source: J Immunol. 2020 Aug 17;205(6):1503–12. doi: 10.4049/jimmunol.2000525 (PMC7477745; doi:10.4049/jimmunol.2000525)
Supplement: Data Supplement [file JI_2000525.zip › JI_2000525_Supplemental_Figures_1.pdf]

Supplementary Information: Human CD8$^+$ T cells exhibit a shared antigen threshold for different effector responses

Enas Abu-Shah[†§⋆], Nicola Trendel[†⋆], Philipp Kruger[†], John Nguyen[†], Johannes Pettmann[†], Mikhail Kutuzov[†], Omer Dushek[†¶]

[†]Sir William Dunn School of Pathology, [§]Kennedy Institute of Rheumatology
University of Oxford.
[⋆]Equal contribution, [¶]Corresponding author

A

Name	Sequence	Dissociation constant	Dissociation rate
9V	SLLMWITQV :	$K_D = 7.07 \times 10^{-5}$ μM	$k_{off} = 8.26 \times 10^{-5}$ s^{-1}
4A	SLLAWITQV :	$K_D = 1.09 \times 10^{-3}$ μM	$k_{off} = 1.54 \times 10^{-3}$ s^{-1}
5F	SLLMFITQV :	$K_D = 9.31 \times 10^{-2}$ μM	$k_{off} = 1.01 \times 10^{1}$ s^{-1}
8K	SLLMWITKV :	$K_D = 2.03 \times 10^{-1}$ μM	$k_{off} = 8.33 \times 10^{-2}$ s^{-1}
5P	SLLMPITQV :	$K_D = 4.85 \times 10^{-1}$ μM	$k_{off} = 9.8 \times 10^{-1}$ s^{-1}
4A8K	SLLAWITKV :	$K_D = 1.78 \times 10^{0}$ μM	$k_{off} = 1.95 \times 10^{0}$ s^{-1}
4A5A	SLLAAITQV :	$K_D = 9.67 \times 10^{1}$ μM	N.D
4A5P8K	SLLAPITKV :	$K_D = 1.40 \times 10^{3}$ μM	N.D

B

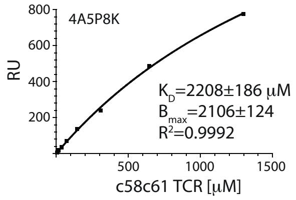

C

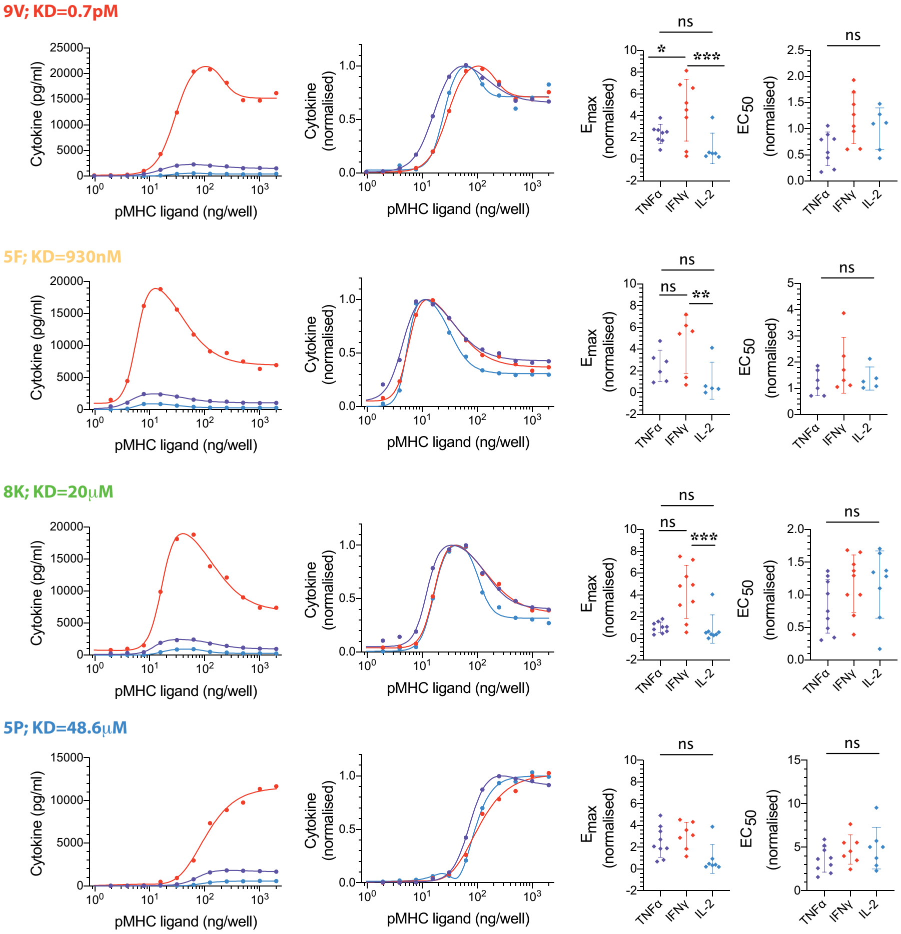

Figure S1: Binding parameters for the pMHC panel and additional functional data A) The dissociation constant and dissociation rate for the 8 pMHC used measured by surface plasmon resonance (SPR) at 37 degrees. All parameters except for 4A5A and 4A5P8K were reported previously (16). B) SPR experiments were repeated for the two lowest affinity pMHC at 37 degrees as previously described (16) with higher TCR concentrations to more accurately determine the dissociation constant. Representative binding response at steady-state over the TCR concentration is shown for 4A5P8K. The average of at least 3 independent experiments were used to calculate the K_D values in panel A. C)Changes to the pMHC affinity does not introduce differences in EC_{50} for each cytokine. Direct comparison of normalised E_{max} (left dotplot) and EC_{50} (right dotplot) for TNFα, IFNγ and IL-2 for different affinity pMHC (rows) showing significant but $<$ 2-fold differences between IFNγ and IL-2 on the one hand and TNFα on the other hand. Error bars are SD of mean for 9 independent donors. Normalisation of experimental data is described in Materials and Methods. ANOVA corrected for multiple comparisons by Tukey's test (*; p=0.0383, **; p=0.006, ***; p<0.001).

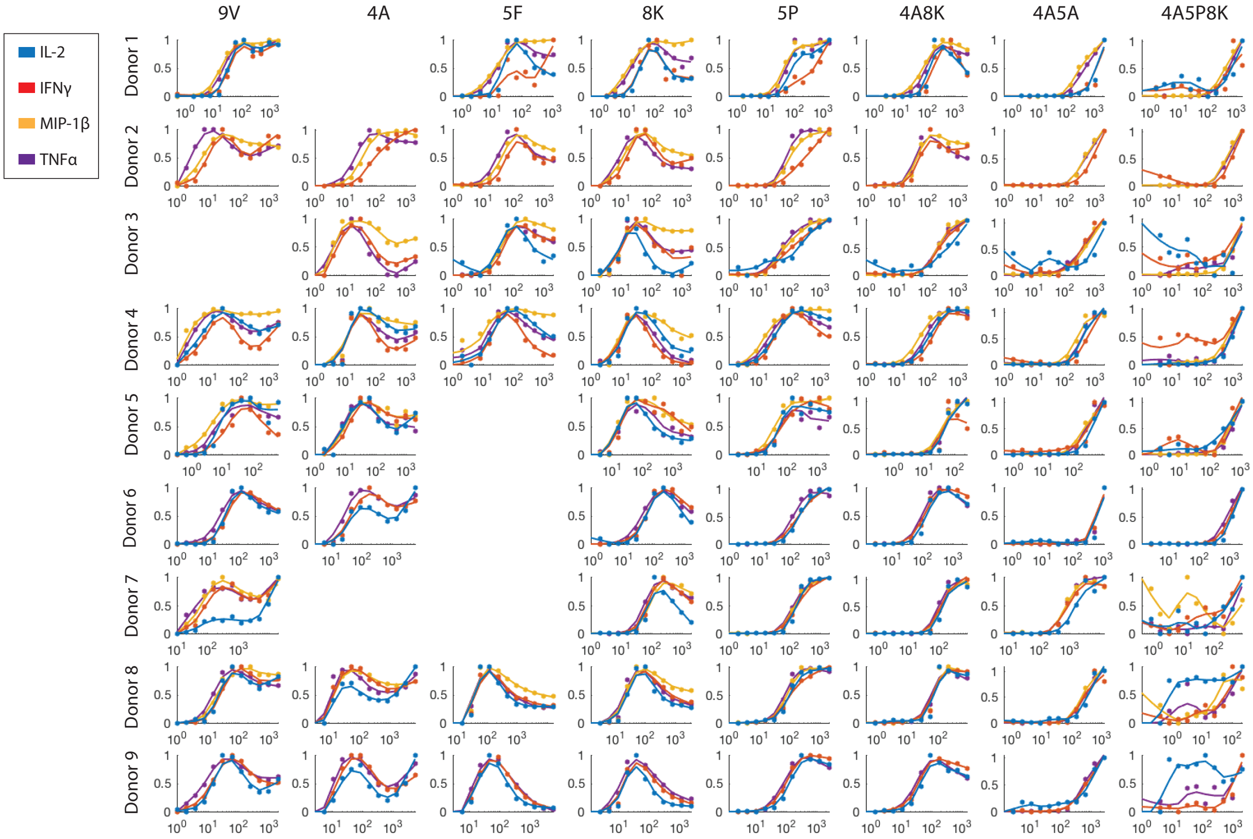

Figure S2: Overlay of cytokine dose-response curves highlights that different cytokines have a comparable antigen dose threshold and this conclusion holds for different antigen affinity. The dose-response curves for TNFα, IFNγ, MIP1β and IL-2 were normalised by their respective E_{max} value for different affinity pMHCs (columns) and individual donors (rows). Although the two lowest affinity pMHCs (4A5A, 4A5P8K) were omitted from the quantitative analysis because accurate estimates of EC_{50} were not possible, representative curves highlight that they also share a common antigen threshold. Additionally, MIP1β exhibited a shared antigen threshold but was omitted from the main text because it was not measured in all experimental conditions. Normalisation of experimental data is described in Materials and Methods.

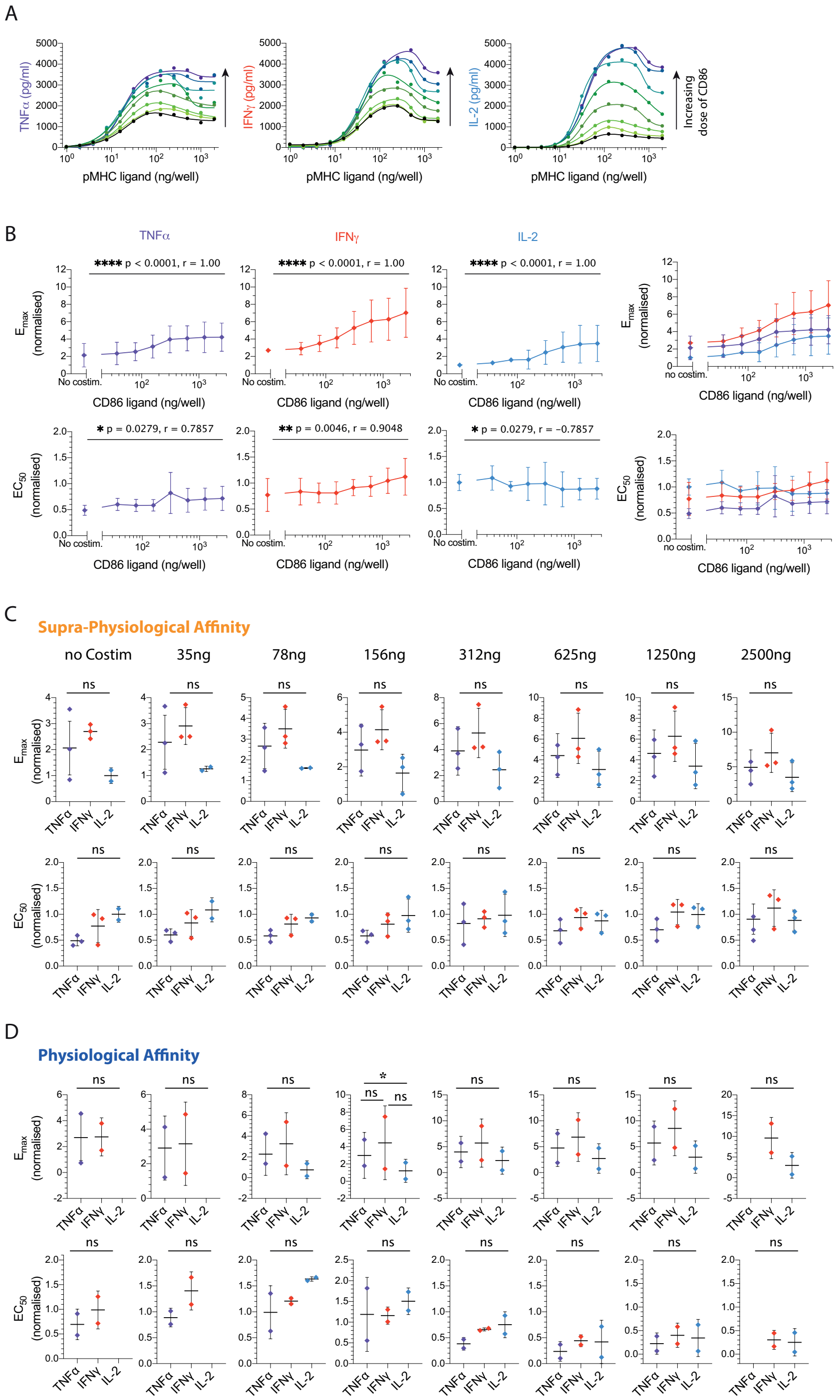

Figure S3: CD28 co-stimulation does not alter the threshold for cytokine secretion. A) Representative dataset showing secretion of TNFα, IFNγ and IL-2 as a function of supra-physiological affinity pMHC dose (4A) when T cells were co-stimulated with different doses of CD86 ligand (colours). B) Top row: Normalised E_{max} of cytokine produced as a function of CD86 dose confirms that co-stimulation amplifies cytokine secretion for individual cytokines or an overlay of all three (right panel). Bottom row: CD86 co-stimulation has a minor impact on the EC_{50} of cytokine secretion in response to the supra-physiological-affinity pMHC mutant. C-D) Direct comparison of normalised E_{max} (top) and EC_{50} (bottom) for TNFα, IFNγ and IL-2 for different doses of CD86 ligand (columns) showing no significant and $<$ 2-fold differences between the three cytokines independent of CD86 co-stimulation for (C) supra-physiological, from the plots in B and (D) physiological-affinity pMHC (D) from plots in Fig. 3. Error bars are SD of mean for 3 independent donors. Normalisation of experimental data is described in Materials and Methods. Solid lines in representative datasets are the fits used to extract E_{max} and EC_{50}. ANOVA corrected for multiple comparisons by Tukey's test (*; p=0.0266).

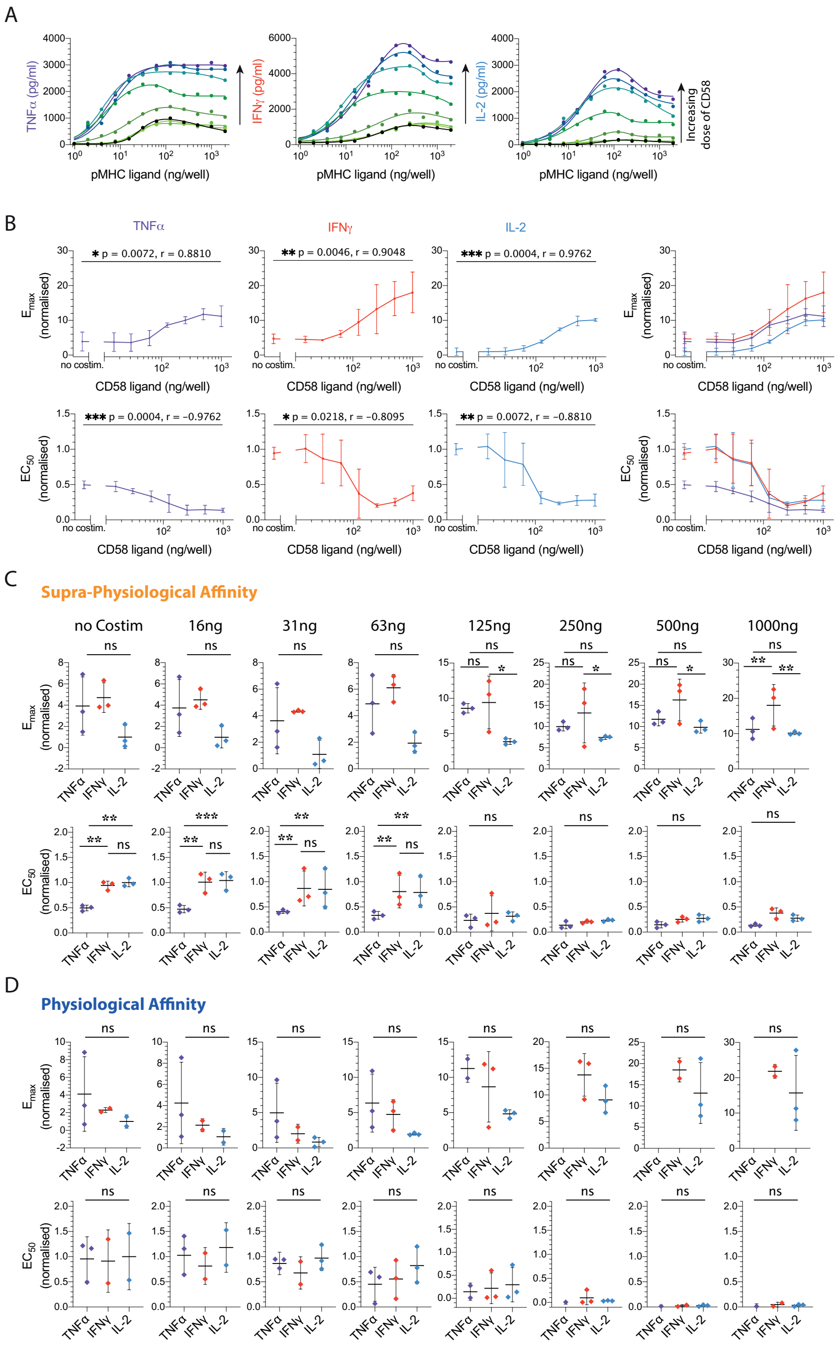

Figure S4: CD2 co-stimulation does not alter the threshold for cytokine secretion. A) Representative dataset showing secretion of TNFα, IFNγ and IL-2 as a function of supra-physiological affinity pMHC (4A) dose when T cells were co-stimulated with different doses of CD58 (colours). B) Top row: Normalised E_{max} of cytokine produced as a function of CD58 dose confirms that co-stimulation amplifies cytokine secretion for individual cytokines or an overlay of all three (right panel). Bottom row: CD58 co-stimulation has a profound impact on the EC_{50} of cytokine secretion in response to the supra-physiological-affinity pMHC mutant. C-D) Direct comparison of normalised E_{max} (top) and EC_{50} (bottom) for TNFα, IFNγ and IL-2 for different doses of CD58 (columns) showing no significant and $<$ 2-fold differences between the three cytokines independent of CD58 co-stimulation for (C) supra-physiological, from the plots in B and (D) physiological-affinity pMHC (D) from plots in Fig. 4. Error bars are SD of mean for 3 independent donors. Normalisation of experimental data is described in Materials and Methods. Solid lines in representative datasets are the fits used to extract E_{max} and EC_{50}. ANOVA corrected for multiple comparisons by Tukey's test (**; p=0.005, ***; p=0.0007).
